# Ninjin’yoeito is associated with attenuation of postoperative decline in Prognostic Nutritional Index in patients with oral squamous cell carcinoma: a single-center, open-label, single-arm study

**DOI:** 10.3389/fnut.2026.1851978

**Published:** 2026-08-14

**Authors:** Hajime Suzuki, Takuya Yoshimura, Haruka Amitani, Koshiro Tagawa, Yuka Hirano, Ryohei Tada, Hirono Migita, Masahiro Tezuka, Takayuki Ishida, Kiyohide Ishihata, Hiroko Watanabe, Masayuki Hirose, Toshitaka Futagawa, Eishi Kuroda, Nanami Sameshima Uto, Marie Amitani, Norifumi Nakamura, Hideto Saijo, Akio Inui

**Affiliations:** 1 Department of Oral and Maxillofacial Surgery, Kagoshima University Graduate School of Medical and Dental Sciences, Kagoshima, Japan; 2 Pharmacological Department of Herbal Medicine, Kagoshima University Graduate School of Medical and Dental Sciences, Kagoshima, Japan; 3 Division of Psychosomatic Internal Medicine, Kagoshima University Graduate School of Medical and Dental Sciences, Kagoshima, Japan; 4 Center for Clinical and Translational Research, Kyushu University Hospital, Fukuoka, Japan; 5 Department of Statistics and Data Center, Clinical Research Support Center Kyushu, Fukuoka, Japan; 6 Department of Pharmacy, Kagoshima University Hospital, Kagoshima, Japan; 7 Division of Community-Based Medicine, Kagoshima University Graduate School of Medical and Dental Sciences, Kagoshima, Japan; 8 Department of Oral and Maxillofacial Surgery, Faculty of Dentistry, Universitas Indonesia, Depok, Indonesia

**Keywords:** Kampo medicine, Ninjin’yoeito, oral squamous cell carcinoma, perioperative care, perioperative nutrition, Prognostic Nutritional Index

## Abstract

**Background:**

Postoperative nutritional deterioration and immunosuppression are clinically important in patients undergoing surgery for oral squamous cell carcinoma (OSCC). The Prognostic Nutritional Index (PNI), calculated from serum albumin and total lymphocyte count, is associated with the prognosis of OSCC. Ninjin’yoeito (NYT), a traditional Japanese Kampo medicine, has been reported to exert orexigenic and immunomodulatory effects. We evaluated whether perioperative NYT administration was associated with attenuation of postoperative PNI decline in patients with OSCC.

**Methods:**

This single-center, open-label, single-arm interventional study was conducted at Kagoshima University Hospital, approved by a Certified Review Board, and registered with the Japan Registry of Clinical Trials (jRCTs071230055). Patients with OSCC scheduled for curative surgery received NYT (7.5 g/day) from the preoperative period through postoperative week 8. The primary endpoint was the change in PNI from baseline to postoperative week 8, compared with a prespecified historical threshold (−4.95) using one-sample *t*-test. Secondary endpoints were summarized descriptively with 95% confidence intervals (CIs).

**Results:**

Nineteen patients completed the study protocol and were included in the efficacy and safety analyses. The mean change in PNI at postoperative week 8 was −2.576 (95% CI, −4.743 to −0.410), which was significantly better than the historical threshold (*p* = 0.0335). The exploratory secondary outcomes, including body weight and selected inflammatory indices, showed postoperative trajectories compatible with gradual recovery during follow-up. No grade ≥3 treatment-related adverse events or serious adverse events were observed. Given the single-arm design with a historical comparator, these findings should be interpreted cautiously and warrant confirmation in controlled trials.

**Conclusion:**

Perioperative NYT administration was associated with attenuation of postoperative PNI decline in patients undergoing OSCC surgery. NYT was well tolerated, supporting the feasibility of its perioperative use. Controlled trials are warranted to confirm clinical benefits and evaluate long-term outcomes of NYT use.

## 1 Introduction

Oral squamous cell carcinoma (OSCC) is a common malignancy of the head and neck. Surgical resection remains the standard curative treatment. Major head and neck cancer surgeries demand complex multidisciplinary perioperative care, and the Enhanced Recovery After Surgery (ERAS) pathways have been promoted to reduce morbidity and complications and improve care efficiency (1). Perioperative nutritional management is a core component of ERAS-based care, and the European Society for Clinical Nutrition and Metabolism (ESPEN) practical guidelines for clinical nutrition in surgery recommend avoiding prolonged fasting, re-establishing oral intake early, and initiating nutritional therapy without delay when nutritional risk becomes apparent to mitigate the catabolic impact of surgical stress (2).

Malnutrition in cancer is multifactorial and associated with poorer quality of life, increased treatment-related toxicities, and adverse clinical outcomes. The ESPEN recommends that nutritional issues be addressed at diagnosis and managed in parallel with anticancer therapy (3). The Prognostic Nutritional Index (PNI), calculated from serum albumin and peripheral lymphocyte count, is a simple composite measure that integrates nutritional and immunological status (4). In patients with OSCC undergoing radical surgery, preoperative PNI has been identified as an independent prognostic indicator of overall survival (OS) and disease-free survival (DFS), with time-dependent receiver operating characteristic analyses suggesting superior prognostic discrimination compared with other systemic inflammatory markers (5). Consistently, in our previous study on aged patients with OSCC, pretreatment PNI demonstrated excellent predictive value for 5-year OS and DFS, underscoring the clinical relevance of PNI-based risk stratification in this population (6). Taken together, these findings support PNI as a clinically meaningful prognostic biomarker in OSCC and motivate the evaluation of perioperative strategies that may help preserve PNI.

Ninjin’yoeito (NYT), a traditional Japanese Kampo medicine comprising 12 crude drugs, has been used clinically to treat symptoms such as fatigue and anorexia (7–9). Mechanistic studies suggest that NYT activates hypothalamic appetite-related pathways, including arcuate nucleus neuropeptide Y neurons via distinct voltage-dependent Ca^2+^ channels and partial mediation through orexin 1 receptor (OX1R) signaling (7, 8). In a cancer cachexia model, NYT attenuated the loss of skeletal muscle function in tumor-bearing mice (9). Clinically, NYT has been evaluated in older adults with frailty, and the feasibility of its co-administration during adjuvant chemotherapy has also been reported (10, 11). However, whether perioperative NYT administration mitigates the postoperative decline in PNI among patients with OSCC undergoing surgery remains unclear. We conducted a single-center, open-label, single-arm interventional study to investigate whether perioperative NYT administration attenuates the postoperative decline in PNI in patients with OSCC undergoing surgery.

## 2 Materials and methods

### 2.1 Study design and participants

This single-center, open-label, single-arm interventional study was conducted at the Kagoshima University Hospital. The study protocol was approved by the Kagoshima University Certified Review Board (CRB7210001) and registered with the Japan Registry of Clinical Trials (jRCTs071230055). Written informed consent was obtained from all participants before enrollment. Eligible participants were adults (≥18 years) with newly diagnosed, previously untreated OSCC scheduled for curative surgical resection under general anesthesia. In addition, participants were required to have at least one of the following conditions: anorexia, fatigue/malaise, or anemia (hemoglobin <14 g/dL in men and <12 g/dL in women), and be able to take the study drug orally (including via tube feeding) within 1 week after surgery. Key exclusion criteria were inability to ingest or absorb food/oral medications, evident cognitive impairment, a planned postoperative course of chemotherapy or radiotherapy, use of Kampo medicines within the preceding 2 weeks, current or planned use of anamorelin, or any condition judged by the investigators to make participation inappropriate. Baseline values were defined as the most recent measurements obtained between the dates of informed consent and screening.

### 2.2 Intervention and perioperative nutritional management

The participants received NYT (Kracie, Ltd., Tokyo, Japan) extract granules at a total daily dose of 7.5 g, divided into two doses administered orally before or between meals. NYT was initiated 2–4 weeks before surgery and continued until 8 weeks after surgery (total planned administration: 10–12 weeks). Periods during which medication intake was not feasible, such as temporary inability to take oral or tube medications, were excluded from the study. Concomitant treatments were generally permitted, except for the following prohibited therapies during the study period: use of Kampo medicines other than NYT, postoperative chemotherapy, and radiotherapy.

Adherence to NYT was assessed at each scheduled visit based on patient interviews. Completion of the planned perioperative NYT administration was defined as continuation of NYT from the preoperative period through postoperative week 8 without permanent discontinuation.

Perioperative nutritional management followed institutional standard practice for patients undergoing surgery for OSCC. Oral intake was resumed once the surgical wound condition had stabilized, typically within 7–14 days after surgery. Until oral intake was resumed, enteral nutrition was provided via a nasogastric tube. Oral nutritional supplements were not routinely used. Albumin infusion was not administered, and no patient required blood transfusion during the perioperative period. Antimicrobial agents were used as clinically indicated.

### 2.3 Outcomes

The primary endpoint was the change in PNI from baseline (before surgery) to 8 weeks after surgery. PNI was calculated using the Onodera’s formula: 10 × serum albumin (g/dL) + 0.005 × total lymphocyte count (/μL).

Secondary endpoints included (i) the change and percentage change in PNI at prespecified time points; (ii) inflammatory markers (e.g., neutrophil-to-lymphocyte ratio [NLR], lymphocyte-to-monocyte ratio [LMR], platelet-to-lymphocyte ratio [PLR], Glasgow Prognostic Score and modified Glasgow Prognostic Score [GPS/mGPS]); (iii) nutritional indices (CONUT score) and biomarkers (short-term nutritional markers such as prealbumin, transferrin, and retinol-binding protein); and (iv) clinical outcomes, including body weight and body mass index (BMI), appetite and fatigue questionnaires (SNAQ and CFS), food intake rate during hospitalization, skin graft survival rate, and postoperative infections, including surgical site infection (SSI).

Study assessments were scheduled at 2–4 weeks preoperatively, on the day of surgery, postoperative days 3, 7, and 14 (as applicable), and at postoperative weeks 4 and 8. For postoperative assessments, an allowable window of ±1 day was applied, and assessments were therefore commonly performed on postoperative days 4, 8, and 15. These visit windows were prespecified to accommodate routine postoperative clinical workflows.

Safety was assessed throughout the study by monitoring adverse events (AEs), performance status (PS), and laboratory tests (hematology and biochemistry). AEs were categorized as skin and subcutaneous tissue disorders (e.g., eczema and rash), gastrointestinal disorders (e.g., nausea and diarrhea), and metabolic and nutritional disorders (e.g., anorexia).

### 2.4 Analysis populations

The efficacy analysis set (EAS) included enrolled patients, excluding those who did not undergo surgery, those who never received the study drug, and those without post-baseline efficacy data after the initiation of NYT. The safety analysis set (SAS) included enrolled patients, excluding those who did not receive the study drug.

### 2.5 Statistical analysis

The sample size was determined using a historical cohort at the Kagoshima University Hospital (34 patients from January 2009 to December 2017). The historical cohort comprised consecutive OSCC patients treated at the same institution between 2009 and 2017 who underwent curative surgery and had PNI assessed at baseline and postoperative week 8. Differences in perioperative practices over time cannot be excluded. In this cohort, the mean change in PNI from baseline to 8 weeks post-surgery was −4.95 with a standard deviation of 5.86. Assuming that NYT would prevent postoperative deterioration in PNI (i.e., mean change = 0), the historical mean (−4.95) was set as the threshold for the primary analysis. With a common standard deviation of 6.5, two-sided significance level of 5, and 80% power, the required sample size was 16 patients for a one-sample *t*-test. Allowing for an approximate dropout rate of 20%, the target sample size was set at 20 patients.

For the primary endpoint, the mean change in PNI at 8 weeks (EAS) was compared against the prespecified historical threshold (−4.95) using a one-sample *t*-test; two-sided *p* < 0.05 was considered statistically significant. The distribution of the primary endpoint change score was assessed using the Shapiro–Wilk test. As a sensitivity analysis for the primary endpoint, a non-parametric Wilcoxon signed-rank test was performed using the historical median change in PNI as the reference value. The mean change and its corresponding two-sided 95% confidence interval (CI) were calculated. For secondary endpoints, analyses were performed in the EAS. First, changes in PNI from baseline to postoperative week 4, as well as percentage changes in PNI from baseline to postoperative weeks 4 and 8, were evaluated using one-sample *t*-tests against prespecified historical threshold means (−5.20, −9.61, and −9.04, respectively), with corresponding two-sided 95% CIs. These historical threshold means were derived from the same institutional historical cohort used for the primary endpoint and corresponded to the mean absolute change in PNI at postoperative week 4, the mean percentage change in PNI at postoperative week 4, and the mean percentage change in PNI at postoperative week 8, respectively. Second, for NLR, LMR, PLR, GPS/mGPS, CONUT, body weight, BMI, SNAQ, prealbumin, transferrin, retinol-binding protein, CFS, and hematology parameters (including total lymphocyte count), absolute and percentage changes from baseline at each time point were calculated, along with time-point–specific summary statistics. Third, food intake rates (breakfast, lunch, dinner, and their arithmetic mean) were analyzed in a similar manner, using postoperative day 4 as the baseline. Finally, skin graft survival and surgical site infection were summarized as frequencies and proportions at each time point. Missing data were not imputed. All analyses were performed using the SAS software (version 9.4; SAS Institute Inc., Cary, NC, USA).

## 3 Results

### 3.1 Patient characteristics and study flow

**Figure 1:**
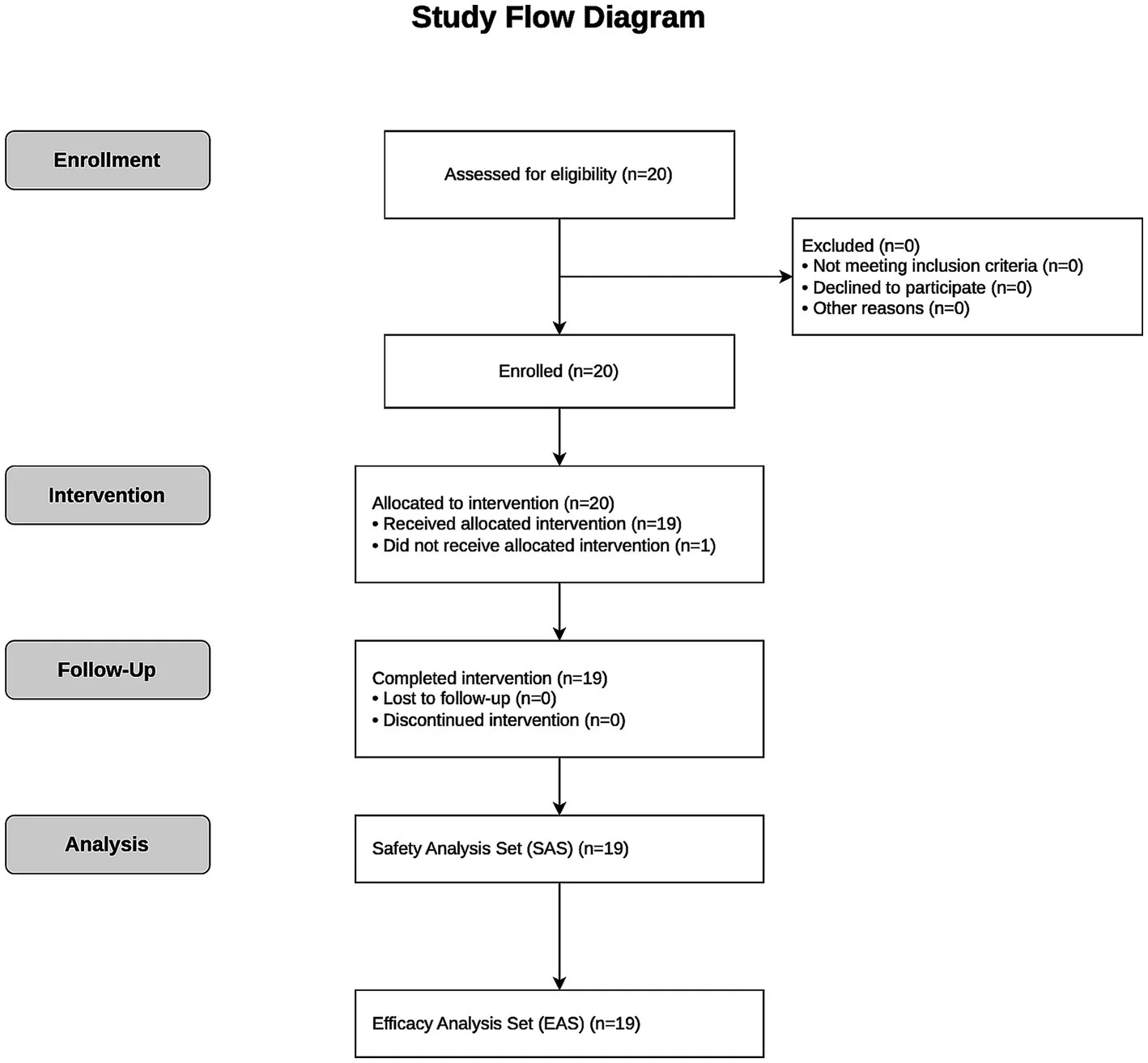
Study flow diagram of patient enrollment, intervention, follow-up, and analysis sets. Twenty patients were enrolled in this study. Nineteen patients received Ninjin’yoeito (NYT), completed the intervention, and were included in both efficacy analysis set (EAS) and safety analysis set (SAS). One enrolled patient did not receive NYT and was excluded from both analysis sets.

A total of 20 patients were enrolled in this study. One patient did not receive the study drug (NYT) and was excluded from the analysis; therefore, 19 patients were included in both the EAS and SAS (Figure 1). Baseline demographic and clinical characteristics of the enrolled patients are summarized in Table 1. Surgical procedures and reconstruction methods for the EAS are provided in Supplementary Table S1. All 19 patients included in the EAS and SAS completed the planned perioperative NYT administration through postoperative week 8. No patient permanently discontinued NYT during the study period.

**Table 1:** Baseline demographic and clinical characteristics of enrolled patients (*n* = 20).

Characteristic	Value
Age, years	67.5 ± 11.1 (median 68.0; range 36–84)
Sex, *n* (%)	Male 11 (55.0); Female 9 (45.0)
Height, cm	160.67 ± 8.54 (median 160.00; range 142.5–175.6)
Weight, kg	61.06 ± 10.84 (median 59.30; range 37.5–78.4)
BMI, kg/m^2^	23.50 ± 2.86 (median 24.07; range 18.5–28.4)
Hemoglobin, g/dL	14.005 ± 1.728 (median 14.200; range 12.00–18.80)
Medical history, *n* (%)	Yes 15 (75.0); No 5 (25.0)
Comorbidity, *n* (%)	Yes 14 (70.0); No 6 (30.0)
Smoking status, *n* (%)	Never 10 (50.0); Former 9 (45.0); Current 1 (5.0)
Primary tumor site, *n* (%)	Tongue 11 (55.0); Maxillary gingiva 2 (10.0); Mandibular gingiva 3 (15.0); Floor of mouth 1 (5.0); Palate 2 (10.0); Lip 1 (5.0); Buccal mucosa 0 (0.0)
Clinical T category, *n* (%)	Tis 3 (15.0); T1 4 (20.0); T2 8 (40.0); T3 3 (15.0); T4a 2 (10.0); T4b 0 (0.0)
Clinical N category, *n* (%)	N0 18 (90.0); N1 1 (5.0); N2b 1 (5.0); N2a/N2c/N3 0 (0.0)
Clinical M category, *n* (%)	M0 20 (100.0); M1 0 (0.0)
Clinical stage, *n* (%)	I 7 (35.0); II 7 (35.0); III 3 (15.0); IVa 3 (15.0); IVb/IVc 0 (0.0)
ECOG performance status, *n* (%)	0: 13 (65.0); 1: 6 (30.0); 3: 1 (5.0); 2/4: 0 (0.0)

Data are shown for all enrolled patients (*n* = 20). One enrolled patient did not receive Ninjin’yoeito and was excluded from the efficacy analysis set (EAS) and safety analysis set (SAS) (*n* = 19).

### 3.2 Primary endpoint

**Figure 2:**
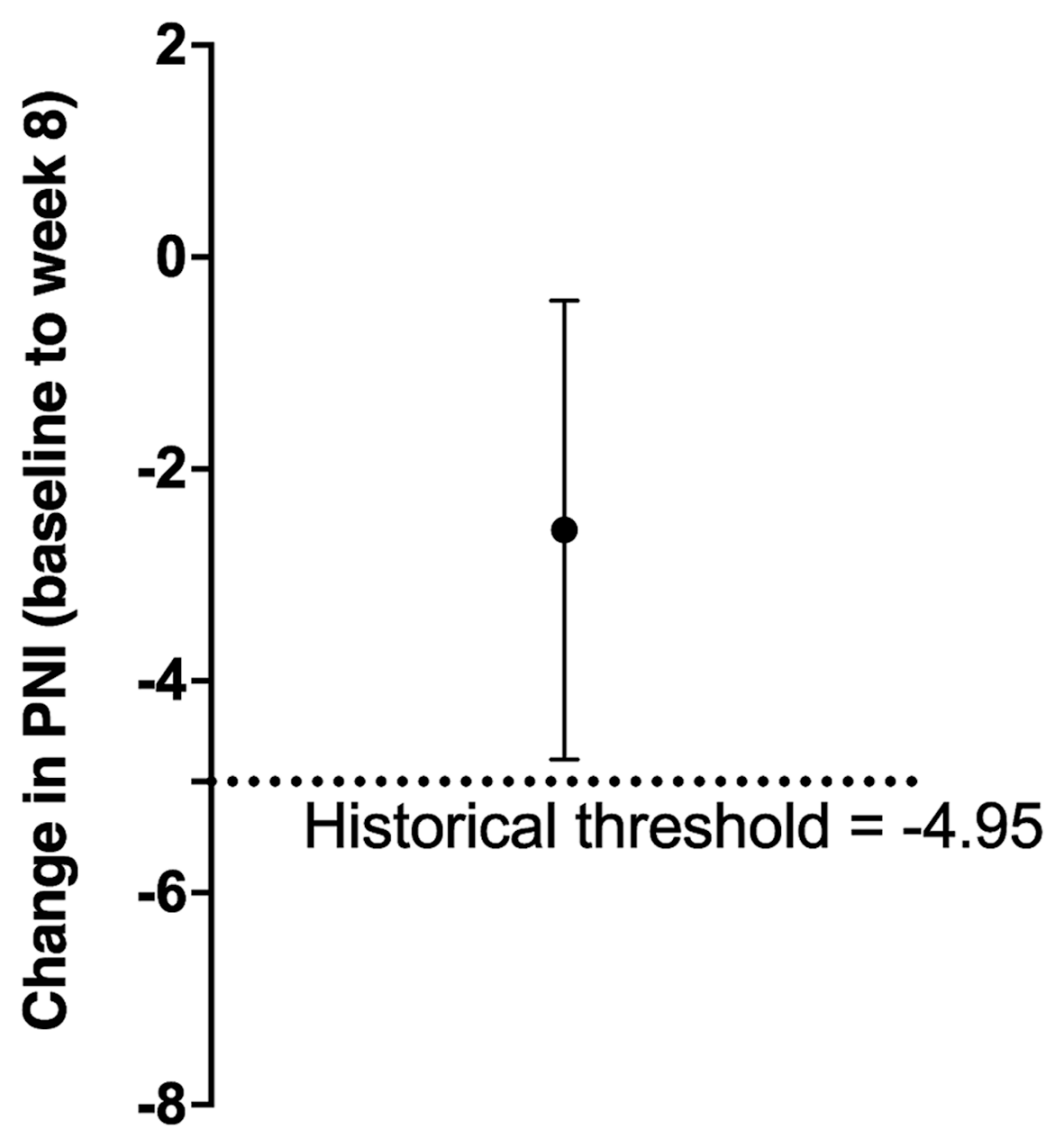
Primary endpoint: change in Prognostic Nutritional Index (PNI) from baseline to postoperative week 8 (EAS, *n* = 19). Dots indicate the mean change, and vertical bars indicate 95% confidence interval (CI). Dashed line denotes the prespecified historical threshold (−4.95). The mean change was −2.576 (95% CI, −4.743 to −0.410), which was significantly better than the threshold based on one-sample *t*-test (*p* = 0.0335).

The primary endpoint was the change in PNI from baseline (before surgery) to 8 weeks after surgery. In the EAS (*n* = 19), the mean change in PNI at 8 weeks was −2.576 (95% CI, −4.743 to −0.410). This value was significantly better than the prespecified historical threshold (mean change, −4.950) based on one-sample *t*-test (*p* = 0.0335) (Figure 2). The time course of PNI is shown in Supplementary Figure S1. The distribution of week-8 PNI change scores did not show evidence of deviation from normality by the Shapiro–Wilk test (*p* = 0.8061). In the non-parametric sensitivity analysis, the Wilcoxon signed-rank test using the historical median change in PNI (−5.5088) as the reference value also showed a significant difference (*p* = 0.0181). PNI values were available for all 19 patients in the EAS at all scheduled assessment time points; therefore, no imputation or sensitivity analysis for missing primary endpoint data was required.

### 3.3 Secondary endpoints

At postoperative week 4, the mean change in PNI was −5.782 (95% CI, −7.727 to −3.836), which was not significantly better than the prespecified historical threshold (−5.20; one-sample *t*-test, *p* = 0.5379). The mean percentage change in PNI at postoperative week 4 was −11.284% (95% CI, −14.896 to −7.672), also not significantly better than the historical threshold (−9.61; *p* = 0.3431). At postoperative week 8, the mean percentage change in PNI was −5.016% (95% CI, −9.267 to −0.765), which was numerically improved relative to the historical threshold (−9.04) but did not reach statistical significance (*p* = 0.0622). Descriptive summaries of other nutritional and inflammatory markers are provided in Table 2 and Supplementary Tables S4 and S7–S9. CONUT scores and short-term nutritional markers, including prealbumin, transferrin, and retinol-binding protein, were not available for all patients at all time points because these measurements were obtained as part of routine clinical practice rather than as mandatory assessments at every visit. Therefore, these endpoints were summarized descriptively and interpreted cautiously.

**Table 2:** Changes in nutritional and inflammatory markers from baseline to postoperative weeks 4 and 8 (EAS, *n* = 19).

Variable	Baseline (screening)	Week 4 (day 29)	Week 8 (day 57)	Change through week 8 (95% CI)*
PNI	50.87 ± 4.17	45.08 ± 4.96	48.29 ± 5.63	−2.58 (−4.74 to −0.41)
Body weight (kg)	61.64 ± 10.81	58.01 ± 9.86	57.94 ± 9.97	−3.70 (−4.84 to −2.56)
BMI (kg/m^2^)	23.71 ± 2.78	22.33 ± 2.52	22.31 ± 2.59	−1.40 (−1.80 to −1.00)
NLR	2.92 ± 1.07	2.31 ± 1.84	2.54 ± 1.50	−0.38 (−1.00 to 0.24)
Total lymphocyte count (/μL)	1657.4 ± 337.4	1637.9 ± 518.9	1721.1 ± 567.9	63.7 (−148.3 to 275.7)
SNAQ	14.7 ± 1.6	15.0 ± 1.4	14.8 ± 1.6	0.2 (−0.8 to 1.1)
CFS	16.5 ± 6.9	17.8 ± 8.1	15.6 ± 8.2	−0.9 (−4.0 to 2.2)

Values are presented as mean ± standard deviation (SD) unless otherwise specified. No multiplicity adjustment was applied. Prespecified one-sample *t*-tests for secondary PNI endpoints were performed against historical thresholds (reported in the Results). Other secondary endpoints are presented descriptively. 95% CIs for changes are calculated from SD of the change scores using the *t* distribution (*n* = 19), without imputation for missing data.

Among patients who underwent forearm free-flap reconstruction with split-thickness skin grafting for donor-site coverage (*n* = 13), graft survival at the donor site was 100% on postoperative day 4 and 92.3% thereafter (postoperative days 8 and 15 and postoperative weeks 4 and 8) (Supplementary Table S2). No cases of SSI were observed on postoperative day 4; SSI occurred in one patient (5.3%) on postoperative day 8 and in two patients (10.5%) on postoperative day 15 and at postoperative week 4, with no cases observed at postoperative week 8 (Supplementary Table S3).

### 3.4 Safety

Safety was evaluated using the SAS (*n* = 19). AEs occurred in 13 patients (68.4%). The most frequent AEs were diarrhea and anorexia (*n* = 7 each), and all AEs were of grade 1–2. No grade ≥3 AEs or serious AEs were reported (Table 3). Routine laboratory parameters are summarized in Supplementary Tables S5 and S6.

**Table 3:** Adverse events (AEs) in the safety analysis set (SAS).

Adverse event	Any grade, *n* (%)	Grade 1, *n*	Grade 2, *n*	Grade ≥3, *n*
Any AE (patients with ≥1 AE)	13 (68.4)	—	—	0
Skin and subcutaneous tissue disorders				
Eczema	2 (10.5)	1	1	0
Maculopapular rash	2 (10.5)	1	1	0
Urticaria	2 (10.5)	1	1	0
Pruritus	1 (5.3)	0	1	0
Gastrointestinal disorders				
Nausea	4 (21.1)	4	0	0
Vomiting	3 (15.8)	3	0	0
Abdominal pain	2 (10.5)	2	0	0
Diarrhea	7 (36.8)	7	0	0
Metabolism and nutrition disorders				
Anorexia	7 (36.8)	7	0	0

Values indicate the number (%) of patients experiencing each AE, and grades are summarized as the worst grade experienced by each patient for each event. No grade ≥3 AEs were observed.

## 4 Discussion

The present single-center, open-label, single-arm study suggests that perioperative administration of NYT may attenuate the postoperative decline in PNI in patients undergoing surgery for OSCC. In the primary analysis, the change in PNI from baseline to postoperative week 8 was significantly better than the prespecified historical threshold. Prespecified secondary analyses for PNI at postoperative week 4 (change and percentage change) and percentage change at week 8 were not powered for hypothesis-driven inference, as the sample size was determined based on the primary endpoint; thus, these analyses did not demonstrate statistical superiority over their respective historical thresholds. However, the observed PNI trajectory suggested a stepwise recovery from early postoperative decline toward week 8. NYT was also well tolerated in the perioperative setting, with no grade ≥3 AEs or serious AEs observed. This finding is clinically relevant because perioperative care in major head and neck cancer surgeries is increasingly organized around standardized pathways that aim to reduce postoperative morbidity while accelerating recovery. The ERAS-based consensus recommendations for major head and neck surgery with free-flap reconstruction emphasize coordinated perioperative management across disciplines (1). In parallel, clinical nutrition guidelines for surgery and cancer populations emphasize the early restoration of oral intake, avoidance of prolonged fasting, and timely escalation of nutritional therapy when nutritional risk becomes apparent (2, 3). Within this context, perioperative intervention is most useful when integrated into routine workflows, has acceptable tolerability, and targets clinically meaningful domains such as immunonutritional status.

Biological plausibility of the observed pattern exists, although the mechanistic inference remains speculative. NYT has been reported to act on appetite-related hypothalamic pathways, including activation of ghrelin-responsive neuronal populations and complementary orexin 1 receptor–mediated effects (7, 8). In addition, NYT attenuated loss of skeletal muscle function in tumor-bearing mice (9), which is consistent with its potential role in counterbalancing catabolic pressure. In our cohort, the postoperative trajectory of oral intake and recovery of nutritional markers were compatible with gradual restoration during follow-up; however, these observations should be interpreted as supportive rather than causal evidence.

PNI also incorporates lymphocyte count, and NYT has been examined in clinical settings associated with frailty, where its multifactorial effects have been considered (10). Case-level clinical experience suggests the feasibility of NYT co-administration even during demanding treatment courses such as postoperative chemotherapy (11). In the current study, lymphocyte-related indices showed postoperative perturbations with subsequent recovery, consistent with the expected perioperative immune dynamics. Determining whether NYT significantly alters these trajectories and identifying the underlying immunological mechanism require controlled studies with prespecified immune endpoints. To place our exploratory clinical outcomes in perspective, evidence from the perioperative immunonutrition literature can be informative but heterogeneous. Meta-analyses in head and neck surgical populations suggest that immunonutrition may reduce selected postoperative complications or improve recovery metrics in some contexts; however, its effects are not uniform across different protocols and settings (12, 13). Therefore, our observations should be viewed as hypothesis-generating rather than as confirmatory evidence, particularly in the absence of a concurrent control group and with a modest sample size.

The clinical rationale for focusing on perioperative PNI is supported by its established prognostic relevance in OSCC. Preoperative PNI has been reported to be an independent prognostic indicator in patients with OSCC undergoing radical surgery (5). In our institutional cohort, a low preoperative PNI was independently associated with worse disease-specific survival in patients with surgically treated OSCC (6). Consistent with these observations, a meta-analysis has reported that low PNI was associated with poorer outcomes in oral cancer (14) and more broadly in head and neck squamous cell carcinoma (15). In this setting, limiting the magnitude and duration of postoperative PNI decline may be a pragmatic supportive target. Thus, our study does not demonstrate an improvement in long-term oncological outcomes, and survival benefits should be evaluated in future controlled trials.

This study has few limitations that should be considered when interpreting the findings. As the sample size calculation was based on the primary endpoint, analyses of secondary endpoints should be interpreted as nominal and exploratory, and no multiplicity adjustment was applied. Although the primary endpoint findings were supported by a non-parametric sensitivity analysis and PNI values were complete at all scheduled time points, the modest sample size and the use of a historical comparator still limit causal inference. First, the single-arm design without a concurrent control group precludes definitive attribution of the observed PNI trajectory to NYT. Although a prespecified historical threshold was used for comparison, changes in perioperative practice over time and unmeasured confounding factors may influence postoperative PNI recovery. Furthermore, the current cohort had a more favorable distribution of T and N categories and clinical stage than the historical cohort. This imbalance may itself have contributed to the smaller observed postoperative decline in PNI, independently of NYT administration. In addition, the open-label nature of the study could affect subjective outcomes such as appetite, although the primary endpoint was derived from objective laboratory values. Second, this was a single-center study with a modest sample size, which limits precision and generalizability across institutions and surgical pathways. Follow-up was confined to the perioperative period; therefore, the study cannot address whether perioperative NYT translates into improvements in long-term oncological outcomes, including recurrence and survival. Finally, the mechanistic interpretation remains limited because immunological endpoints and comprehensive nutritional assessments were not prespecified as primary mechanistic outcomes. Future randomized controlled trials with standardized perioperative nutritional protocols and longer follow-up periods are warranted to confirm whether perioperative NYT improves clinically meaningful outcomes, including postoperative complications and survival.

## 5 Conclusion

In conclusion, perioperative administration of NYT was associated with attenuation of the postoperative decline in PNI in patients undergoing surgery for OSCC compared with a prespecified historical threshold. NYT was well tolerated, with no grade ≥3 treatment-related AEs or serious AEs observed. These findings support feasibility and justify controlled trials to determine whether perioperative NYT improves clinically meaningful outcomes.

## Data Availability

The summary data supporting the conclusions of this article are included in the article and Supplementary Material. Individual-level clinical data are not publicly available due to ethical and privacy restrictions; de-identified data may be provided upon reasonable request with appropriate institutional approvals.
