# Supplementary material for: Ninjin’yoeito is associated with attenuation of postoperative decline in Prognostic Nutritional Index in patients with oral squamous cell carcinoma: a single-center, open-label, single-arm study
Source: Front Nutr. 2026 Aug 14;13:1851978. doi: 10.3389/fnut.2026.1851978 (PMC13521862; doi:10.3389/fnut.2026.1851978)
Supplement: Supplementary file 1 [file Table_1.DOCX]

Supplementary Material

**Supplementary Figure S1. Time course of the Prognostic Nutritional Index (PNI) from screening to postoperative week 8 (EAS, n=19).**

Values are mean ± SD at each time point (screening; postoperative days 4, 8, and 15; postoperative week 4 [day 29]; and postoperative week 8 [day 57]). No hypothesis testing was performed for this exploratory time-course visualization.

**Supplementary Table S1. Surgical procedures and reconstruction methods (EAS, n=19).**

| **Surgical category** | **Reconstruction method** | **n (%)** |
| --- | --- | --- |
| Primary tumor resection alone | None (primary closure) | 6 (31.6) |
| Primary tumor resection + unilateral neck dissection | Free flap | 11 (57.9) |
| Primary tumor resection + bilateral neck dissection | Free flap | 2 (10.5) |

Presented for the Efficacy Analysis Set (EAS, n=19). Percentages are based on n=19.

Reconstruction categories indicate the main reconstructive procedure. Reconstruction categories indicate the main reconstructive procedure. Split-thickness skin grafting for coverage of the forearm flap donor site is summarized separately in Supplementary Table S2.

**Supplementary Table S2. Survival of split-thickness skin grafts used for forearm flap donor-site coverage (EAS, n=13).**

| **Time point** | **Assessed, n** | **Graft survival, n (%)** |
| --- | --- | --- |
| Postoperative day 4 | 13 | 13 (100.0) |
| Postoperative day 8 | 13 | 12 (92.3) |
| Postoperative day 15 | 13 | 12 (92.3) |
| Postoperative week 4 (day 29) | 13 | 12 (92.3) |
| Postoperative week 8 (day 57) | 13 | 12 (92.3) |

Split-thickness skin grafts were harvested from the abdomen and used to cover the forearm free-flap donor site; graft survival was assessed at the donor site.

**Supplementary Table S3. Surgical site infection (SSI) over time (EAS, n=19).**

| **Time point** | **Assessed, n** | **SSI, n (%)** |
| --- | --- | --- |
| Postoperative day 4 | 19 | 0 (0.0) |
| Postoperative day 8 | 19 | 1 (5.3) |
| Postoperative day 15 | 19 | 2 (10.5) |
| Postoperative week 4 (day 29) | 19 | 2 (10.5) |
| Postoperative week 8 (day 57) | 19 | 0 (0.0) |

**Supplementary Table S4. Food intake rate during the early postoperative period (EAS, n=19).**

| **Meal / Summary** | **Statistic** | **POD4** | **POD8** | **POD15** | **POD22 (3 weeks)** |
| --- | --- | --- | --- | --- | --- |
| **Breakfast intake (%)** | Mean ± SD | 57.9 ± 50.7 | 91.6 ± 23.4 | 88.9 ± 25.8 | 91.1 ± 21.8 |
| Median | 100.0 | 100.0 | 100.0 | 100.0 |  |
| Range | 0–100 | 0–100 | 0–100 | 10–100 |  |
| **Lunch intake (%)** | Mean ± SD | 52.6 ± 51.3 | 86.8 ± 31.3 | 79.5 ± 36.1 | 90.0 ± 23.3 |
| Median | 100.0 | 100.0 | 100.0 | 100.0 |  |
| Range | 0–100 | 0–100 | 0–100 | 10–100 |  |
| **Dinner intake (%)** | Mean ± SD | 63.2 ± 49.6 | 82.1 ± 34.7 | 77.9 ± 37.8 | 97.4 ± 6.5 |
| Median | 100.0 | 100.0 | 100.0 | 100.0 |  |
| Range | 0–100 | 0–100 | 0–100 | 80–100 |  |
| **Arithmetic mean of three meals (%)** | Mean ± SD | 57.9 ± 45.6 | 86.8 ± 24.5 | 82.1 ± 29.4 | 92.8 ± 15.6 |
| Median | 66.7 | 100.0 | 100.0 | 100.0 |  |
| Range | 0–100 | 0–100 | 0–100 | 33–100 |  |

- Food intake rate was calculated for each meal as a percentage of the served amount consumed.
- The arithmetic mean represents the simple average of breakfast, lunch, and dinner intake rates.
- No hypothesis testing was performed; values are presented descriptively.

**Supplementary Table S5. Hematology parameters over time (SAS, n=19).**

|  | Screening | POD4 | POD8 | POD15 | Week 4 (day29) | Week 8 (day57) |
| --- | --- | --- | --- | --- | --- | --- |
| RBC (×10^6/µL) | 4.617±0.732 | 3.959±0.481 | 3.941±0.459 | 3.732±0.585 | 4.008±0.387 | 4.238±0.594 |
| Hemoglobin (g/dL) | 14.068±1.751 | 12.111±1.226 | 11.953±1.051 | 11.368±1.595 | 12.011±0.914 | 12.484±1.336 |
| Hematocrit (%) | 42.82±4.74 | 36.89±3.74 | 36.50±3.72 | 34.81±5.24 | 37.34±2.77 | 39.06±4.27 |
| WBC (×10^3/µL) | 6.915±1.570 | 10.251±3.992 | 9.283±4.616 | 10.292±13.756 | 5.604±1.950 | 6.118±1.517 |
| Neutrophils (%) | 66.89±7.84 | 78.23±12.02 | 68.93±12.03 | 67.80±10.88 | 57.86±9.88 | 60.97±11.47 |
| Eosinophils (%) | 2.58±3.82 | 1.64±1.75 | 3.15±2.03 | 2.81±2.18 | 4.81±4.33 | 3.54±3.90 |
| Basophils (%) | 0.61±0.24 | 0.31±0.22 | 0.57±0.23 | 0.57±0.27 | 0.71±0.28 | 0.55±0.17 |
| Monocytes (%) | 5.18±1.60 | 5.46±1.72 | 6.41±1.87 | 5.59±1.78 | 5.91±1.83 | 5.67±1.48 |
| Lymphocytes (%) | 24.74±5.70 | 14.37±10.09 | 20.93±11.13 | 23.23±9.96 | 30.72±8.61 | 29.27±10.17 |
| Platelets (×10^3/µL) | 247.8±77.6 | 207.7±64.9 | 307.8±92.7 | 458.1±224.7 | 240.6±91.5 | 236.9±70.8 |

Values are mean ± SD. POD indicates postoperative day; RBC, red blood cell count; WBC, white blood cell count.

The large SD for WBC at POD15 reflects a transient extreme value in one patient, which was verified against the source medical record. This value was considered clinically attributable to a temporary infection.

**Supplementary Table S6. Blood biochemistry parameters over time (SAS, n=19).**

|  | Screening | POD4 | POD8 | POD15 | Week 4 (day29) | Week 8 (day57) |
| --- | --- | --- | --- | --- | --- | --- |
| Albumin (g/dL) | 4.26±0.36 | 2.98±0.54 | 3.20±0.55 | 3.35±0.49 | 3.69±0.30 | 3.97±0.37 |
| Total bilirubin (mg/dL) | 0.87±0.39 | 0.84±0.40 | 0.63±0.23 | 0.55±0.12 | 0.74±0.78 | 0.64±0.18 |
| AST (U/L) | 28.8±9.6 | 34.2±21.9 | 47.2±37.2 | 24.5±8.0 | 22.2±7.3 | 21.3±7.9 |
| ALT (U/L) | 27.4±17.5 | 36.3±30.9 | 65.3±63.0 | 35.2±21.1 | 22.4±10.6 | 16.9±11.7 |
| LDH (U/L) | 181.3±21.8 | 183.1±38.9 | 202.2±46.8 | 173.7±48.4 | 148.9±17.5 | 164.5±24.1 |
| ALP (U/L) | 76.0±20.0 | 77.9±25.1 | 107.3±57.4 | 91.7±39.1 | 77.8±20.6 | 73.5±18.1 |
| γ-GTP (U/L) | 57.9±52.6 | 77.6±66.6 | 145.5±178.4 | 92.9±113.0 | 46.9±45.0 | 32.9±23.9 |
| CRP (mg/dL) | 0.191±0.281 | 11.893±8.986 | 5.349±5.129 | 1.143±2.070 | 0.399±0.627 | 1.312±4.042 |
| BUN (mg/dL) | 14.30±4.13 | 12.65±4.60 | 17.92±4.85 | 17.58±10.07 | 12.22±3.26 | 14.28±4.50 |
| Creatinine (mg/dL) | 0.826±0.203 | 0.643±0.156 | 0.673±0.117 | 0.755±0.150 | 0.782±0.176 | 0.759±0.148 |
| Sodium (mEq/L) | 140.2±2.8 | 141.2±2.9 | 139.1±2.4 | 138.6±2.9 | 140.3±2.1 | 139.7±3.5 |
| Potassium (mEq/L) | 4.11±0.36 | 3.94±0.31 | 4.04±0.40 | 4.53±0.42 | 4.21±0.38 | 4.19±0.38 |

Values are mean ± SD. POD indicates postoperative day; AST, aspartate aminotransferase; ALT, alanine aminotransferase; LDH, lactate dehydrogenase; ALP, alkaline phosphatase; γ-GTP, gamma-glutamyl transpeptidase; CRP, C-reactive protein; BUN, blood urea nitrogen.

**Supplementary Table S7. Inflammatory ratios over time (EAS, n=19).**

| **Variable** | **Statistic** | **Screening** | **POD4** | **POD8** | **POD15** | **Week 4 (day29)** | **Week 8 (day57)** |
| --- | --- | --- | --- | --- | --- | --- | --- |
| NLR | n | 19 | 19 | 19 | 19 | 19 | 19 |
|  | Mean ± SD | 2.92±1.07 | 9.23±6.86 | 4.65±3.20 | 4.70±6.89 | 2.31±1.84 | 2.54±1.50 |
|  | Median (range) | 2.61 (1.5–5.6) | 7.55 (1.6–27.0) | 4.19 (1.2–14.1) | 3.47 (1.1–32.7) | 1.60 (1.2–9.1) | 1.88 (0.8–6.3) |
| LMR | n | 19 | 19 | 19 | 19 | 19 | 19 |
|  | Mean ± SD | 5.13±1.78 | 2.75±2.04 | 3.64±2.67 | 4.57±2.82 | 5.79±2.84 | 5.55±2.39 |
|  | Median (range) | 4.66 (2.7–8.7) | 2.24 (0.7–7.7) | 2.34 (1.4–10.9) | 3.69 (1.4–10.7) | 5.36 (1.7–12.5) | 6.04 (1.5–10.0) |
| PLR | n | 19 | 19 | 19 | 19 | 19 | 19 |
|  | Mean ± SD | 151.68±46.42 | 199.19±75.91 | 205.32±66.84 | 293.11±142.12 | 160.78±80.66 | 151.23±66.69 |
|  | Median (range) | 155.78 (71.4–248.5) | 189.45 (64.1–395.9) | 192.08 (89.4–304.9) | 301.55 (72.7–569.1) | 139.68 (53.2–396.5) | 135.62 (78.1–298.9) |

NLR, neutrophil-to-lymphocyte ratio; LMR, lymphocyte-to-monocyte ratio; PLR, platelet-to-lymphocyte ratio. POD indicates postoperative day.

**Supplementary Table S8. Inflammatory and nutritional scores over time (EAS).**

| **Variable** | **Statistic** | **Screening** | **POD4** | **POD8** | **POD15** | **Week 4 (day29)** | **Week 8 (day57)** |
| --- | --- | --- | --- | --- | --- | --- | --- |
| GPS | n | 19 | 19 | 19 | 19 | 19 | 19 |
|  | Mean ± SD | 0.1±0.2 | 1.8±0.4 | 1.4±0.9 | 0.8±0.7 | 0.4±0.6 | 0.2±0.4 |
|  | Median (range) | 0.0 (0–1) | 2.0 (1–2) | 2.0 (0–2) | 1.0 (0–2) | 0.0 (0–2) | 0.0 (0–1) |
| mGPS | n | 19 | 19 | 19 | 19 | 19 | 19 |
|  | Mean ± SD | 0.1±0.2 | 1.8±0.4 | 1.4±0.9 | 1.2±0.8 | 0.4±0.6 | 0.3±0.5 |
|  | Median (range) | 0.0 (0–1) | 2.0 (1–2) | 2.0 (0–2) | 1.0 (0–2) | 0.0 (0–2) | 0.0 (0–1) |
| CONUT score | n | 16 | 14 | 14 | 15 | 14 | 16 |
|  | Mean ± SD | 1.1±0.9 | 5.4±3.1 | 3.4±2.3 | 3.1±2.4 | 1.9±1.4 | 1.3±1.5 |
|  | Median (range) | 1.0 (0–3) | 6.5 (0–11) | 3.0 (0–8) | 3.0 (0–9) | 1.5 (1–6) | 1.0 (0–5) |

CONUT scores were not available for all patients at all time points because total cholesterol measurements were obtained as part of routine clinical practice and were not mandatory at every visit.

GPS, Glasgow Prognostic Score; mGPS, modified Glasgow Prognostic Score; CONUT, Controlling Nutritional Status. POD indicates postoperative day.

**Supplementary Table S9. Short-term nutritional markers over time (EAS).**

| **Marker** | **Unit** | **Statistic** | **Screening** | **POD15** | **Week 4 (day29)** | **Week 8 (day57)** |
| --- | --- | --- | --- | --- | --- | --- |
| Prealbumin | mg/dL | n | 16 | 8 | 9 | 7 |
|  |  | Mean ± SD | 28.01±4.57 | 27.10±3.69 | 24.32±4.83 | 26.13±4.92 |
|  |  | Median (range) | 29.15 (18.3–35.2) | 27.40 (22.1–31.4) | 25.30 (14.4–30.8) | 25.20 (18.3–32.8) |
| Transferrin | mg/dL | n | 16 | 8 | 9 | 7 |
|  |  | Mean ± SD | 245.6±43.6 | 224.4±28.6 | 211.9±31.7 | 239.4±43.3 |
|  |  | Median (range) | 252.5 (177–308) | 220.0 (187–265) | 209.0 (179–279) | 253.0 (176–302) |
| Retinol-binding protein | mg/dL | n | 16 | 8 | 9 | 7 |
|  |  | Mean ± SD | 3.34±0.50 | 3.38±0.74 | 2.82±0.60 | 2.87±0.72 |
|  |  | Median (range) | 3.40 (2.2–4.0) | 3.70 (2.3–4.1) | 3.00 (1.7–3.7) | 2.80 (2.0–4.0) |

Short-term nutritional markers were obtained as part of routine clinical practice and were not mandatory at every visit; therefore, the number of available observations varied across time points.

Short-term nutritional markers were not assessed at POD4 and POD8. POD indicates postoperative day.

**Supplementary Table S10. Comparison of baseline demographic and clinical characteristics between the current NYT cohort and the historical cohort.**

| **Characteristic** | **Current NYT cohort (enrolled patients, n=20)** | **Historical cohort (n=34)** |
| --- | --- | --- |
| Age, years | 67.5 ± 11.1 (median 68.0; range 36–84) | 69.0 ± 13.6 (median 71.0; range 29.0–86.0) |
| Sex, n (%) | Male 11 (55.0); Female 9 (45.0) | Male 21 (61.8); Female 13 (38.2) |
| Height, cm | 160.67 ± 8.54 (median 160.00; range 142.5–175.6) | 158.0 ± 8.7 (median 161.2; range 139.8–172.3) |
| Weight, kg | 61.06 ± 10.84 (median 59.30; range 37.5–78.4) | 57.6 ± 11.7 (median 55.0; range 40.2–89.9) |
| BMI, kg/m² | 23.50 ± 2.86 (median 24.07; range 18.5–28.4) | 23.0 ± 3.8 (median 22.4; range 15.4–32.7) |
| Hemoglobin, g/dL | 14.005 ± 1.728 (median 14.200; range 12.00–18.80) | 14.0 ± 1.7 (median 13.9; range 10.0–16.5) |
| Medical history, n (%) | Yes 15 (75.0); No 5 (25.0) | Yes 32 (94.1); No 2 (5.9) |
| Comorbidity, n (%) | Yes 14 (70.0); No 6 (30.0) | Yes 24 (70.6); No 10 (29.4) |
| Smoking status, n (%) | Never 10 (50.0); Former 9 (45.0); Current 1 (5.0) | Never 15 (44.1); Former 10 (29.4); Current 9 (26.5) |
| Primary tumor site, n (%) | Tongue 11 (55.0); Maxillary gingiva 2 (10.0); Mandibular gingiva 3 (15.0); Floor of mouth 1 (5.0); Palate 2 (10.0); Lip 1 (5.0); Buccal mucosa 0 (0.0) | Tongue 11 (32.4); Maxillary gingiva 5 (14.7); Mandibular gingiva 11 (32.4); Floor of mouth 4 (11.8); Palate 1 (2.9); Lip 0 (0.0); Buccal mucosa 2 (5.9) |
| Clinical T category, n (%) | Tis 3 (15.0); T1 4 (20.0); T2 8 (40.0); T3 3 (15.0); T4a 2 (10.0); T4b 0 (0.0) | Tis 0 (0.0); T1 5 (14.7); T2 10 (29.4); T3 11 (32.4); T4a 8 (23.5); T4b 0 (0.0) |
| Clinical N category, n (%) | N0 18 (90.0); N1 1 (5.0); N2b 1 (5.0); N2a/N2c/N3 0 (0.0) | N0 13 (38.2); N1 7 (20.6); N2b 7 (20.6); N2a/N2c/N3 7 (20.6) |
| Clinical M category, n (%) | M0 20 (100.0); M1 0 (0.0) | M0 34 (100.0); M1 0 (0.0) |
| Clinical stage, n (%) | I 7 (35.0); II 7 (35.0); III 3 (15.0); IVa 3 (15.0); IVb/IVc 0 (0.0) | I 3 (8.8); II 4 (11.8); III 9 (26.5); Iva 18 (52.9); IVb/IVc 0 (0.0) |
| ECOG performance status, n (%) | 0: 13 (65.0); 1: 6 (30.0); 3: 1 (5.0); 2/4: 0 (0.0) | 0: 23 (67.6); 1: 3 (8.8); 2: 8 (23.5); 3: 0 (0.0); 4: 0 (0.0) |

Values are shown as mean ± SD (median; range) or n (%). The current cohort includes all enrolled patients (n=20); one enrolled patient did not receive NYT and was excluded from the EAS and SAS. The historical cohort comprised patients with OSCC treated at Kagoshima University Hospital between January 2009 and December 2017.
